# A cross‐sectional study on the ability of physicians to hypertension management in China's Sichuan Tibetan rural area

**DOI:** 10.1111/jch.14351

**Published:** 2021-08-21

**Authors:** Runyu Ye, Xin Zhang, Zhipeng Zhang, Xiangyu Yang, Xiaoping Chen

**Affiliations:** ^1^ Department of Cardiology West China Hospital Sichuan University Chengdu China

**Keywords:** clinical practice, hypertension, knowledge, primary care, training

## Abstract

This study aimed to investigate the hypertension management abilities of rural physicians in a high‐altitude Tibetan area. A cross‐sectional survey was conducted in the Ganzi Tibetan Autonomous Prefecture, China, in October 2020. Information about healthcare resources in local medical institutions, along with the knowledge, attitudes, practices, and training status of primary care physicians, was collected. Limited resources were observed in terms of equipment, drugs, and personnel in the 18 township hospitals included. A total of 132 physicians participated in this survey. The scores for hypertension‐related knowledge, attitudes toward hypertension management, routine practice ability, priority given to hypertension, and confidence in performing certain tasks were 32.60%, 67.40%, 18.90%, 65.15%, and 35.60%, respectively. The most concerning issues lay in the ignorance of the healthy lifestyle, undervaluation of cardiovascular risks, and lack of confidence in optimally performing management activities. Only 9.85% of the physicians received more than 24 days of training per year; 28.79% preferred a longer training time. While training was generally provided in conference sessions (63.64% of current training programs), physicians preferred remote education (55.30%), and on‐site guidance (46.21%) from professionals. The current training was centered around clinical skills (61.36%) and was identified as a major training requirement by the physicians surveyed (80.30%). This survey suggests that the medical resources may not be effective, with deficiencies present in the knowledge and practices of primary care physicians in the Sichuan Tibetan area. Hypertension education and skill‐development courses based on the specific issues identified should be provided to these physicians in the future.

## 1 INTRODUCTION

Hypertension is the main risk factor for cardiovascular and cerebrovascular diseases and all‐cause deaths worldwide.^1^, ^2^ There are around 245 million hypertensive patients in China, which is a heavy burden on families and society.^3^ Primary care health institutions (community health service centers, community health service stations, township hospitals, and village clinics) are the main battlefield of hypertension management in China; thus, the level of hypertension management in these institutions directly affects the trend of cardiovascular and cerebrovascular disease development.^4^ In 2009, a national policy called Essential Public Health Services (EPHS) was launched in China.^5^ Chronic disease screening and management were provided free of charge to residents by grassroots health institutions.^6^ Studies have shown that the national EPHS program helped improve the quality of hypertension treatment situation.^7^, ^8^ Some studies reported a greater improvement in hypertension management in rural and poorer regions than in urban areas^9^, ^10^; however, others indicated that the urban areas achieved better hypertension management,^11^, ^12^ suggesting that the urban‐rural disparities in the management of hypertension may have persisted.

However, there remains an obvious gap in the economic and medical resources in rural areas with different geographical locations. The per capita gross domestic product (GDP) of the second‐largest Tibetan residential region in China, the Ganzi Tibetan Autonomous Prefecture, which has an average altitude of 3500 m and a population of 1.6 million people, is less than half of the national per capita GDP.^13^, ^14^ The economic and medical resources here lags far behind that of other rural areas. The quality of hypertension management in this region is lower than in urban communities and other rural regions; the region has a higher disease prevalence (32.2% vs. 23.2%) and lower treatment (41.3% vs. 45.8%) and control rates (3.2% vs. 16.8%) for hypertension than the national average.^3^, ^15^ Further, the control rate for hypertension in this region is far less than the rural average control rate (13.1%).^3^ However, most of the previous studies conducted on the management of chronic diseases in urban and rural areas in China focus on areas located in the plains,^10^, ^16^, ^17^ ignoring the situation in Tibetan areas of the plateau.^18^

In this study, we investigated the local healthcare resources and the ability of physicians to adequately manage hypertension in a high‐altitude Tibetan area in Southwest China. We evaluated the hypertension‐related knowledge, attitudes, and primary care practices of these physicians, along with the training requirements to provide education and skill‐development courses for hypertension management in the future.

## 2 METHODS

### 2.1 Study design

A cross‐sectional survey was conducted for this study in October 2020. We identified 132 rural physicians working in 18 township hospitals in Luhuo County, Ganzi Tibetan Autonomous Prefecture. Considering the limited number of physicians, all the identified physicians were invited to participate in our study. Information on local healthcare resources was collected by interviewing each township hospital. A questionnaire was developed according to the basic requirements of the guidelines on the management of hypertension in primary healthcare in China.^4^ We used the Knowledge, Attitudes, and Practices (KAP) survey, which was developed by the World Hypertension League, to test the KAP of health care physicians.^19^ This survey could be used to identify areas where physicians require further education and training to enhance knowledge, attitudes, and skills and improve hypertension management.^19^ The survey was modified to include questions based on the Chinese hypertension guidelines^4^, ^20^ that were translated to Chinese; it was subsequently revised by two professionals to improve the quality of the translation. For evaluation of the training status and requirements, we used the “Questionnaire of Current Status of the Chinese Rural Doctors”.^21^

The professional in charge of the KAP survey translation was appointed as the unified investigator. Before the investigation, we trained a local physician in interpreting the Chinese version of the questionnaire in Tibetan. Subsequently, we gathered the local physicians in a large conference room and handed out the questionnaires. Instructions on how to perform the survey were provided by the unified investigator, translated by the trained Tibetan physician to ensure consistency in terms of quality. Primary care physicians were allowed 40 min to finish the survey independently. Incomplete surveys were checked out on the spot and required to be completed.

The KAP survey contained the following information: (1) demographic characteristics, (2) knowledge of Chinese hypertension guidelines, (3) hypertension management knowledge, (4) attitudes toward hypertension management, (5) practices status during routine hypertension management activities, (6) the priority given to hypertension management tasks, and (7) the confidence in performing hypertension management tasks.^19^

The “Questionnaire of Current Status of the Chinese Rural Doctors” survey included information on the current status and requirements for an annual training program, along with questions on the current and preferred training method and content.^21^

After the survey, two trained medical professionals entered the data in an Excel (Microsoft Excel 2019, Microsoft Corp., Redmond, USA) sheet and conducted statistical analysis, which was reviewed by another professional for accuracy. A medical professional was responsible for data review, and referred to the original data for confirmation or correction if there was any missing or inaccurate information.

The study was conducted in accordance with the principles of the Declaration of Helsinki guidelines,^22^ and all procedures were approved by the Biomedical Research Ethics Committee of the West China Hospital of Sichuan University in Sichuan, China. All participants provided written informed consent.

### 2.2 Related definition

#### 2.2.1 Practicing physician certification and assistant practicing physician certification

In 1999, China implemented a national physician qualification examination system. Physicians are required to pass the national examinations to acquire the practicing physician or the assistant practicing physician certification, which is a recognition of the physician's medical skills. Physicians with assistant practicing physician certification can provide medical care at a village clinic or township hospital independently, or under the guidance of a certified physician at a higher‐level institution.^23^

#### 2.2.2 Hypertension at high altitudes

In this study, the diagnostic standard for chronic hypertension at high altitudes for rural Tibetans was defined as 140/90 mm Hg for systolic/diastolic blood pressure, according to the epidemic studies conducted in Tibetan areas.^20^, ^24^

### 2.3 Statistical analysis

Continuous variables are expressed as mean ± standard deviation (SD). Categorical variables are presented as frequencies (percentages). To summarize the knowledge, attitude, priority, practice, and confidence aspects of the KAP survey, we calculated the score for each part as the number of correct or desirable answers divided by the total score obtained for each part, presented as median percentage (P25, P75). The total score for each section was 100. The chi‐square test was used to compare the differences between the training status and requirements of the rural physicians. Statistical analysis was performed using SPSS version 23.0 (IBM Corp., Armonk, NY, USA), and statistical significance was set at *p* < .05.

## 3 RESULTS

### 3.1 Healthcare resources characteristics

Eighteen township hospitals were included, covering a population of 47 710 people, with a total of 132 rural physicians. The local physician‐to‐population ratio was 2.77 physicians per 1000 people. Of the enrolled institutions, 58.8% were equipped with validated electronic equipment for hypertension measurements. Nearly three quarters of the institutions were equipped with blood and urine test analyzers. None of these hospitals were equipped with a 24‐h ambulatory blood pressure monitoring device; the equipment for hypertension‐related target organ damage, including cardiac ultrasound (11.1%) and arteriosclerosis detectors (0%), was rarely available (Table 1).

**TABLE 1:** Information of basic health‐care resources

Medical equipment available	Category	Number (*N* = 18)
	Mercury sphygmomanometer	17 (94.4%)
	Validated electronic device	13 (58.8%)
	Blood routine analyzer	11 (61.1%)
	Urine routine analyzer	13 (72.2%)
	Blood biochemical analyzer	13 (72.2%)
	Electrocardiogram	15 (83.3%)
	Soft ruler	18 (100%)
	Height and weight meter	18 (100%)
	24‐h ABPM device	0 (0%)
	Cardiac ultrasound	2 (11.1%)
	Arteriosclerosis detector	0 (0%)
Anti‐hypertensive drugs available	agents	Number (*N* = 18)
ACEI	Captopril	14 (77.8%)
ARB	Irbesartan	4 (22.2%)
β blocker	–	0 (0%)
CCB	Nifedipine	12 (66.7%)
	Felodipine	1 (5.6%)
Diuretic	Indapamide	5 (27.8%)
	Hydrochlorothiazide	2 (11.1%)
Single‐pill combination	Irbesartan/ Hydrochlorothiazide	1 (5.6%)
	Reserpine	5 (27.8%)
	Reserpine/Triamterene Hydrochlorothiazide/Dihydralazine	2 (11.1%)

Intermediate‐acting or short‐acting antihypertensive agents were generally prescribed, with captopril and nifedipine sustained‐release tablets being the most common. Additionally, traditional combination preparations, such as reserpine (27.8%) and reserpine/triamterene hydrochlorothiazide/dihydralazine (“Fu‐fang‐li‐xue‐ping‐an‐ben‐die‐ding‐pian”; 11.1%) remained in use. Only one institution administered a new single‐pill combination, such as irbesartan/hydrochlorothiazide, to their patients (Table 1, Supplementary Material 1).

### 3.2 Demographic information

A total of 132 respondents were included in our study (mean age 33.98±8.37 years; 38.6% men; 7.3 physicians per hospital); 39.4% and 47.0% of these had a junior high school or junior college diploma, respectively; only 4.5% had a college degree. Those with practicing physician certification and assistant practicing physician certification accounted for 18.9% and 25.8% of all respondents, respectively (Table 2).

**TABLE 2:** Respondent characteristics

Characteristics	Overall (*N* = 132)
Age (years)	33.98±8.37
Male (%)	51 (38.6%)
Working years	
<5 year	23 (17.4%)
> = 5 year	109 (82.6%)
Education	
College or more	6 (4.5%)
Junior college	62 (47.0%)
Junior high	52 (39.4%)
Lower than junior high school	12 (9.1%)
Specialty	
Clinical medicine	57 (43.2%)
Preventive medicine	23 (17.4%)
General practitioner	29 (22.0%)
Others	23 (17.4%)
Qualification	
Practicing physician certification	25 (18.9%)
Assistant practicing physician certification	34 (25.8%)

### 3.3 Knowledge, attitudes, and practices of primary care physicians

The overall KAP survey results and the detailed answers to each question are presented in Table 3 and Supplementary Material 2, respectively.

**TABLE 3:** Score summary of knowledge, attitude, practice, priority, and confidence among physicians

Survey category	Correct/desired response scoreMedian (P25, P75)
Knowledge	32.60 (16.70, 55.65)
Attitude	67.40 (36.95, 85.20)
Practice	18.90 (9.40, 36.40)
Priority	65.15 (47.38, 74.03)
Confidence	35.60 (33.30, 38.60)

#### 3.3.1 Knowledge

The knowledge score for hypertension management was low as 32.60%. Of all the participants, only 36.4% had learned about the Chinese Hypertension Guidelines, and 17.4% had received training in the guidelines within 2 years previously. Less than 20% of the physicians correctly answered questions regarding the epidemiology knowledge of hypertension, and tended to overestimate the prevalence, awareness, treatment, and control rates for the disease. A total of 72.0% and 58.3% of the physicians knew that 140 mm Hg of systolic blood pressure (SBP) and 90 mm Hg of diastolic blood pressure (DBP) were the thresholds to establish a diagnosis of hypertension. Approximately, 70.5% and 68.2% of the physicians knew that an SBP less than 140 mm Hg and a DBP less than 90 mm Hg could be considered as controlled. Half of the participants knew the recommended salt consumption for lifestyle intervention. The physicians lacked knowledge about the physical activity, weight loss, and alcohol consumption intervention; only 14.4–39.4% of the physicians accurately answered detailed questions on these interventions.

#### 3.3.2 Attitude

The overall attitude toward hypertension management was desirable, with an average score of 67.40%. However, 8.3% of the physicians answered that it was better not to use any drugs or as few drugs as possible even if that meant hypertension was not under control. 13.6% and 31.1% of the physicians considered that antihypertensive agents should be prescribed only if patients were unwilling to make lifestyle changes, or if they were able to make lifestyle changes, respectively. A large proportion of physicians (90%) believed that the blood pressure target and treatment plan should be supported by the patients, and that lifestyle intervention guidance should be provided to all patients.

The majority of physicians (53.0–84.8%) supported sequentially adding drugs to control hypertension, and the use of affordable, high‐quality, long‐acting agents, with combination therapy, and simple, single‐pill agents that were agreed by the patient.

When asked if it is acceptable/desired that a non‐physician healthcare professional could perform certain tasks, approximately one‐third of the physicians supported blood pressure measurement and lifestyle intervention consultation; Only 13.6% believed that non‐physicians could assess cardiovascular risk, and prescribe or modify antihypertensive regimens based on an approved pathway or algorithm.

The physicians believed that the common issues faced in hypertension management optimization were the lack of quality medications (45.5%), adequate training (53.8%) or a clinical team (54.6%), and limited skills in clinical decision making (54.5%) or counseling (50.8%). Some physicians also stated that the patients did not prioritize hypertension management (52.2%).

#### 3.3.3 Practice

The average score for optimal routine practice was low (18.90%). A majority of the participants (75.8%) responded that they used validated electronic blood pressure measurement devices, with 19.7% using mercury devices. For cardiovascular risk assessment, 7.6% of the physicians used a risk calculator, with 64.4% calculating this risk based on their clinical judgment. Several physicians (62.9%) used a paper registration system for people with hypertension who visited their clinics; 18.2% had a computerized registration system, and 18.9% responded that they did not keep a hypertension registry. Further, 68.1% of participants reported keeping a record of patients who were diagnosed with hypertension, and missed blood pressure measurements or a hypertension management appointment.

Of the physicians surveyed, 21.2% performed blood pressure measurement routinely for hypertension screening for 61–90% of routine visits. Approximately one‐third of the physicians provided hypertension consultation to 61–90% of their adult patients to explain the adverse effects of the disease, need for treatment, and home blood pressure measurement device‐usage. Less than 10% of physicians reported that they provided hypertension counselling, such as explaining the usage of a home blood pressure device, or cardiovascular risk assessment for 91–100% of hypertensive people.

Half of the physicians reported prescribing anti‐hypertensive agents for 91–100% of hypertensive people. Of all the participants, 23.5% assessed drug adherence and provided consultation for drug therapy for 61–90% of patients at all visits. Approximately one‐third of physicians would assess cardiovascular risk and prescribe drug treatment based on the risk for 61–90% hypertensive patients, while more than 60% of physicians were among the undesirable group. The physicians had similar responses for lifestyle intervention counselling, such as suggesting regular physical activity and having a healthy body weight (36.4% desirable responses vs. 25.0% undesirable responses). Nearly 60% of the responses pertaining to salt and alcohol intake were undesirable, with only 23.5% and 35.6% of the responses, respectively, being desirable.

For people with an SBP or DBP of 160 mm Hg or 100 mm Hg or more, respectively, 75.0% undesirable responses indicated physicians would prescribe drug therapy for less than 60% of the patients; none of them would prescribe drug therapy for 91–100% of patients, which was the desired response. For those with more than 30% 10‐year cardiovascular event risk, 15.1% and 65.2% of the responses were desirable and undesirable, respectively. For those with a 10‐year cardiovascular disease risk of 20–29%, 9.0% and 73.5% of the responses were desirable and undesirable, respectively. For different high‐risk conditions (established diabetes, prior heart attack, prior stroke, chronic kidney disease, aortic aneurysm, left ventricular hypertrophy, or heart failure), the desirable response rates were 4.5–30.3%, whereas the undesirable response rates were 31.1–65.2%.

In case of various levels of hypertension, a majority of the physicians intended to choose desirable or shorter than recommended follow‐up intervals; 2.3–28.0% of them preferred a longer‐than‐desirable follow‐up duration. However, when presented with a hypertensive emergency or urgency, immediate referral to a hospital was not recommended by 12.1% and 28.8% of physicians, respectively.

#### 3.3.4 Priority

The priority score for hypertension management compared with other routine clinical activities was 65.15%. A majority of the physicians (more than 60%) considered the following aspects as high priorities: accurate diagnosis and counseling for hypertension, its adverse effects, the need for treatment, counseling about drug therapy and adherence to drugs, cardiovascular risk assessment, target blood pressure achievement; and intervention recommendations to the patients for unique barriers in treatment adherence. In terms of cardiovascular risk, 5.3% of the physicians would assess cardiovascular risk for all hypertensive patients, and 7.6% prioritized using an objective tool such as a computer program and calibrating it to the Chinese population.

#### 3.3.5 Confidence

The average score for confidently performing optimal hypertension management activities was low (35.60%). One in three physicians suggested that they were sufficiently confident to prescribe three or more antihypertensive regimes in a single patient, with 57.6% confident in prescribing two drug agents. Without additional training, 33.3% indicated that they could optimally use a hypertension registry, and 18.9% would confidently use a treatment algorithm or pathway. Confidence in perfectly completing routine tasks in diagnosing and managing hypertension without training was 23.5–40.2%. The physicians were least confident about assessing patients’ adherence to antihypertensive drug therapy (23.5%).

### 3.4 Training status and requirements

#### 3.4.1 Current training status and future training requirements

In the previous year, 78.03% of the participants had received less than 12 days of training; 9.85% had received more than 24 days of training. Regarding the method of training, the most common training format was conference sessions (63.64%), followed by remote or video education (39.39%), and self‐education or proficiency tests (29.55%). Clinical skills (61.36%), preventive healthcare knowledge (53.03%), and medication knowledge (43.94%) were most taught in the training sessions, in that order.

In terms of training time requirement, 46.21%, 25.00%, and 28.79% of the physicians indicated that the average training time should be less than 12 days, 12–24 days and more than 24 days per year, respectively. Remote or video education from senior professionals (55.30%) and on‐site guidance from senior professionals (46.21%) seemed to be the most popular training formats, followed by clinical further education (42.42%). Most physicians (80.30%) preferred to receive clinical skills training, while 75.76% of them were also interested in preventive healthcare knowledge (Table 4).

**TABLE 4:** Comparison of current training status and future training requirements

Training content	Training status	Training requirements	*p* value
Training time received annually, days/year^a^			
<12	103 (78.03%)	61 (46.21%)	.000**
12–24	16 (12.12%)	33 (25.00%)	.634
> 24	13 (9.85%)	38 (28.79%)	.006**
Training method			
Conference sessions	84 (63.64%)	51 (38.64%)	.000**
Guidance from senior doctors	22 (16.67%)	61 (46.21%)	.000**
Clinical further education/visiting	18 (13.64%)	56 (42.42%)	.001**
Self‐education or proficiency test	38 (28.79%)	34 (25.76%)	.022*
School training	27 (20.45%)	26 (19.70%)	.063
Remote/video education	52 (39.39%)	73 (55.30%)	.000**
Training content			
Clinical skills	81 (61.36%)	106 (80.30%)	.000**
Medication Knowledge	58 (43.94%)	63 (47.73%)	.000**
Preventive health	70 (53.03%)	100 (75.76%)	.000**

^a^ “ < 12″ and “ > 24″ was used as the basis for the demarcation referred to Ref. [21].

* *p* < .05.

** *p* < .01.

#### 3.4.2 Difference between training status and requirements

The differences between the current training status and future training requirements were statistically significant (Table 4).

Regarding the training time, 12.12% and 9.85% of the physicians received 12–24 days and more than 24 days of training per year, respectively; however, 53.79% preferred a training time of 12 days or longer. Meanwhile, the percentage of those who received less than 12 days of training decreased from 78.03% to 46.21%.

The demand for guidance from professionals, clinical further education, and remote/video education increased markedly, particularly in terms of guidance from senior doctors (16.67% vs. 46.21%). The highest discrepancy between the requirement and current status was for the conference sessions (66.67% vs. 38.64%); self‐education or proficiency tests (25.76%) and school training (19.70%) were the least preferred training methods.

A higher demand was observed for training in clinical skills, preventive health knowledge, and medication knowledge than that currently provided, suggesting that the training provided was not sufficient for the physicians that were surveyed.

## 4 DISCUSSION

In this study, we investigated the medical resources related to hypertension management in the Sichuan Tibetan area, along with the KAP and training status of the physicians. Our results indicate that the hypertension management abilities of physicians in these areas require significant improvement; this partly explains the low hypertension control rate in these poor areas.

The implementation of the EPHS reforms in 2009 has been effective for public health development, with public health services being accessible to more people in China.^10^ However, inequality in hypertension management has persisted. The quality of hypertension management correlates to many factors, including social attention, medical resources, and patients’ involvement.^16^ Compared with residents living in urban or rich regions, those living in poor or rural areas are less likely to be aware of their health conditions or receive timely treatment or blood pressure management.^16^ Most residents in remote areas can only seek medical treatment locally, which limits their access to high‐quality medical resources. Therefore, it is important to improve the quality of chronic disease management in remote areas.

According to the national clinical practice guidelines on the management of hypertension in primary healthcare in China,^4^ it is necessary for primary health institutions to have basic hypertension management equipment and a supply of essential drugs. However, we found that none of these primary institutions met the basic requirements for hypertension management. A serious shortage of talent still exists in the area, with an average of 7.3 physicians per hospital. On an average, these physicians are educated below the junior college education level, and are not qualified with practicing physician certification. The local physician‐to‐population ratio is 2.77 physicians per 1000 people, as opposed to 5.0 per 1000 people for rural regions across the country and in Sichuan,^25^ suggesting a lack of local medical resources.

Adherence to the Chinese hypertension guidelines is key for high‐quality primary care hypertension management; however, physicians in China's Tibetan areas were not sufficiently trained in these guidelines. To our knowledge, this is the first study in China to use the KAP scale to evaluate primary care physicians’ hypertension management ability. The following physician‐related issues in hypertension management were highlighted based on our survey: the physicians (1) neglected the importance of a healthy lifestyle and methods to behavioral and lifestyle changes; (2) lacked confidence to optimally perform routine activities for hypertension management; and (3) underestimated the cardiovascular risks associated with blood pressure; they did not effectively prescribe antihypertensive drugs based on the cardiovascular risk at the individual level. In the future, we plan to create programs for these physicians with an emphasis on hypertension treatment and management, along with establishing a communication system between the Tibetan physicians and hypertension professionals working at tertiary hospitals,^26^ aiming at improving the physicians’ abilities and confidence in terms of hypertension management.

Despite the physicians’ poor educational background, the training currently provided was not effective for the physicians’ chronic disease management abilities. Some physicians were aware of the problem, and were willing to undergo further training to improve their management abilities. However, the current training methods are not tailored to the requirements of the local physicians. We believe that these physicians require a long‐acting and practice‐focused training approach that contrasts with the current conference sessions and tests. Therefore, we should invest more resources in modifying the teaching modes for hypertension management training.

This study did not analyze the reasons for the low level of knowledge and practice for hypertension management, but we believe that this might be associated with the remoteness of the area examined, poor access to high‐quality medical resources, and lack of well‐trained physicians. The results of the physicians’ hypertension management abilities in this study are much poorer than those of physicians in middle‐ and high‐income countries.^27^, ^28^ A previous study from Mongolia^27^ suggested that physicians tended to prioritize hypertension management activities, overlooked the importance of a healthy lifestyle, and lacked the ability to treat various high‐risk patients. These results indicate that most physicians tend to neglect lifestyle modifications and cardiovascular risk assessment. However, the overall hypertension management ability was higher for physicians in Mongolia than the rural physicians in our study, with their scores for the general knowledge, attitude, practice, priority, and confidence for hypertension management being 53.1%, 76.5%, 31.0%, 85.7%, and 63.2%, respectively. Studies have shown that patients in rural China experience poorer hypertension management than those in urban regions.^11^, ^12^ While several studies on hypertension management focus on physicians’ job satisfaction,^29^ and the patients’ trust in them^30^; few studies focus on the physicians’ management abilities. Some studies conducted in rural China^31^, ^32^ investigated the chronic disease management abilities of rural doctors using a self‐made questionnaire. Compared with our study, the physicians in those studies were better aware of the hypertension diagnosis threshold, initiation of drug therapy, and cardiovascular risk assessment; however, they had a similar poor practice ability in providing lifestyle consultation, suggesting poor clinical knowledge and insufficient competence in terms of hypertension management. However, due to the different research scales, we could not accurately compare the differences in management ability between our rural physicians in high‐altitude regions and those situated in the plains. From this perspective, our study fills in the gaps in the field by using the KAP survey developed by the World Hypertension League. The poor hypertension management ability of physicians, limited medical resources, together with the natural environmental characteristics,^15^ contributed to the poor hypertension management quality as our previous study suggested in this high‐altitude region, which was mainly characterized by a high disease prevalence and a relatively low control rate.^15^

Previously, our team had launched a hypertension management improvement project.^26^ We developed an Internet‐based hypertension management system, the Red Shine Chronic Disease Management System (RSCDMS), aiming at providing patients with high‐quality management by physicians under the guidance of specialists to help these physicians improve their clinical disease management abilities.^26^ We hypothesized that by using this system to address the issues identified above, online training courses could be provided based on the physicians’ needs, and a real‐time hierarchical medical system could be created for the high‐altitude remote areas.

This study has several limitations. First, we only included physicians from a county in the Sichuan Tibetan region, which created a selection bias. In the future, we plan on trying to get more funding support to help use a multi‐stage, stratified, random sampling method to enroll the participants in the Tibetan areas, and conducting the survey in the urban community health service centers and rural township hospitals in the plain regions of Sichuan Province. Second, there may be a response bias, as some physicians might have preferred to choose the “obvious” right answers or randomly chosen one option, yet the current answers could still reflect the limited management ability here.

## 5 CONCLUSIONS

This study suggests a lack of effective medical resources, and the deficiencies in the knowledge and practices of primary care physicians in the Sichuan Tibetan area. In the future, long‐term guidance relationships must be built between professionals in tertiary hospitals and physicians at high altitudes, so that training courses can be provided based on the specific issues identified in this study.

## CONFLICT OF INTEREST

None declared.

## AUTHORS CONTRIBUTIONS

Xiaoping Chen is the principal investigator of this study, and designed the study. Runyu Ye and Xin Zhang contributed equally to the review paper, who developed the study concept and wrote the manuscript. Zhipeng Zhang and Xiangyu Yang collected data, designed the method for statistical analysis and participated in the modification of the manuscript. Runyu Ye also was responsible for data reviewing and giving advice on data analysis. All authors reviewed and approved the final manuscript.

## Supporting information

Supporting information

Supporting information
