# Supplementary material for: A cross‐sectional study on the ability of physicians to hypertension management in China's Sichuan Tibetan rural area
Source: J Clin Hypertens (Greenwich). 2021 Aug 21;23(9):1802–9. doi: 10.1111/jch.14351 (PMC8678753; doi:10.1111/jch.14351)
Supplement: Supplementary file 1 — Supporting information [file JCH-23-1802-s001.docx]

**Supplementary Material 1**

**The information of antihypertensive agents in each township hospital**

| Institution | Anti-hypertensive drugs available |
| --- | --- |
| Township hospital 1 | Captopril, Nifedipine, Indapamide, Hydrochlorothiazide |
| Township hospital 2 | Nifedipine |
| Township hospital 3 | Captopril, Nifedipine |
| Township hospital 4 | Captopril, Reserpine,  Reserpine/Triamterene Hydrochlorothiazide/Dihydralazine |
| Township hospital 5 | Captopril, Nifedipine |
| Township hospital 6 | Captopril, Reserpine |
| Township hospital 7 | Captopril, Nifedipine, Reserpine |
| Township hospital 8 | Captopril, Nifedipine, Indapamide |
| Township hospital 9 | Irbesartan |
| Township hospital 10 | Captopril, Nifedipine |
| Township hospital 11 | Captopril, Nifedipine,  Irbesartan/ Hydrochlorothiazide,  Indapamide, Hydrochlorothiazide,  Reserpine/Triamterene Hydrochlorothiazide/Dihydralazine |
| Township hospital 12 | Reserpine |
| Township hospital 13 | Captopril, Nifedipine |
| Township hospital 14 | Captopril, Irbesartan, Indapamide, Reserpine, Reserpine/Triamterene Hydrochlorothiazide/Dihydralazine |
| Township hospital 15 | Captopril, Nifedipine |
| Township hospital 16 | Captopril, Nifedipine |
| Township hospital 17 | Irbesartan |
| Township hospital 18 | Captopril, Irbesartan, Nifedipine, Felodipine, Indapamide |
