# Supplementary material for: A cross‐sectional study on the ability of physicians to hypertension management in China's Sichuan Tibetan rural area
Source: J Clin Hypertens (Greenwich). 2021 Aug 21;23(9):1802–9. doi: 10.1111/jch.14351 (PMC8678753; doi:10.1111/jch.14351)
Supplement: Supplementary file 2 — Supporting information [file JCH-23-1802-s002.docx]

**Supplementary Material 2**

**Responses to the individual questions on the Knowledge, Attitude, Practice, Priority and Confidence**

**Knowledge (Table 1-5)**

**Table 1 Questions Regarding Chinese Hypertension Guidelines**

|  | **Correct/desired answer** | **Correct (%)** | **Incorrect (%)** |
| --- | --- | --- | --- |
| Have you learned about the Chinese Hypertension Guideline? | Yes | 48 (36.4%) | 84 (63.6%) |
| If you have received education/training on the Chinese Hypertension Guidelines when was your most recent training? | Within the last 2 years | 23 (17.4%) | 109 (82.6%) |
| Do you use the Chinese Hypertension Guidelines in all your hypertensive patients? | 8-10 (from scale of 1 to 10, 10 being ‘Always’) | 17 (35.4%) | 31 (64.6%) |

**Table 2** **Questions Regarding Epidemiology Knowledge of Hypertension**

|  | **Correct Answer** | **Correct (%)** | **Underestimated (%)** | **Overestimated (%)** |
| --- | --- | --- | --- | --- |
| What percent of adults in China have hypertension? | 21-30% (or 27.9%) | 22 (16.7%) | 0 (0%) | 110 (83.3%) |
| Of those with hypertension what percent of people are ‘not aware’ they have hypertension or are ‘undiagnosed’? | 41-50% (or 48.4%) | 12 (9.1%) | 75 (56.8%) | 45 (34.1%) |
| Of those with hypertension, what percent of people are ‘not treated’ with antihypertensive medication? | 51-60% (or 54.2%) | 22 (16.7%) | 92 (69.7%) | 18 (13.6%) |
| Of those with hypertension, what percent of people are controlled? | 11-20% (or 16.8%) | 13 (9.9%) | 0 (0%) | 119 (90.2%) |

**Table 3 Questions Regarding General Knowledge of Hypertension**

|  | **Correct Answer** | **Correct (%)** | **Underestimated (%)** | **Overestimated (%)** |
| --- | --- | --- | --- | --- |
| What is the lowest level of usual systolic blood pressure you consider to be ‘hypertensive’ in adults? | 140 mmHg | 95 (72.0%) | 23 (17.4%) | 14 (10.6%) |
| What is the lowest level of usual diastolic blood pressure you consider to be ‘hypertensive’ in most adults adults? | 90 mmHg | 77 (58.3%) | 33 (25.0%) | 22 (16.7%) |
| Lower than what usual level of systolic blood pressure represents blood pressure control in most adult patients? | 140 mmHg | 93 (70.5%) | 26 (19.7%) | 13 (9.8%) |
| Lower than what usual level of diastolic blood pressure represents blood pressure control in most adult patients? | 90 mmHg | 90 (68.2%) | 19 (14.4%) | 23 (17.4%) |

**Table 4** **Questions Regarding General Knowledge of Hypertension**

|  | **Correct/**  **desired answer** | **Correct (%)** | **More aggressive than recommended** | **Less aggressive than recommended** |
| --- | --- | --- | --- | --- |
| What is the recommended daily level of salt consumption for people who have hypertension? | <6g/day | 70 (53%) | 4 (3%) | 58 (43.9%) |
| It is recommended that people with hypertension obtain at least _____ minutes of moderate physical activity most days a week | 30 min | 38 (28.8%) | 7 (5.3%) | 87 (65.9%) |
| If a patient who is overweight and hypertensive loses 1 kilogram weight, what is the average reduction in blood pressure | 0.5-2 mmHg per kg | 52(39.4%) | 68(51.5%) | 12 (9.1%) |
| It is recommended that men with hypertension consume alcohol equal to or less than _______ ml per day. | 50ml | 19 (14.4%) | 13 (9.8%) | 100 (75.8%) |
| It is recommended that women with hypertension consume alcohol equal to or less than _______ml per day. | 25ml | 24 (18.2%) | 18 (13.6%) | 90 (73.2%) |
| How many antihypertensive drugs do most people with hypertension require to achieve blood pressure control? | Two or more | 33 (25.0%) | 82 (62.1%) | 17 (12.9%) |

**Table 5 Questions Regarding General Knowledge of Hypertension**

|  | **Physicians, n (%)** |
| --- | --- |
| **Are there special circumstances where you use a lower blood pressure target?** |  |
| No special target | 19 (14.4%) |
| Diabetes mellitus | 43 (32.6%) |
| Ischemic heart disease | 46 (34.8%) |
| Chronic kidney disease with proteinuria (>1 g/day) | 59 (44.7%) |
| Prior heart attack | 47 (35.6%) |
| Cardiovascular risk more than 20% | 63 (47.7%) |
| Prior stroke | 37 (28.0%) |

**Attitude (Table 6-7)**

**Table 6** **Questions Regarding Attitude**

|  | **Correct/Desired Answer** | **Correct (%)** | **Incorrect (%)** |
| --- | --- | --- | --- |
| Many people with hypertension in the community are not diagnosed. What is the best mechanism for detecting hypertension in the people in your community who do not see health care professionals? | Ticks all the answers  (pharmacies, workplaces, market or trade center) | 24 (18.2%) | 108 (81.8%) |
| **Please indicate if the following statements about controlling hypertension are true or false for most of your hypertension patients** | | | |
| It is better to use no drugs or as few as possible drugs even if that means hypertension is not controlled? | No | 121 (91.7%) | 11(8.3%) |
| The patient should agree with the blood pressure target and treatment plan | Yes | 121 (91.7%) | 11 (8.3%) |
| Drug therapy should only be used if the patient is willing to change their lifestyle | No | 91 (68.9%) | 41 (31.1%) |
| Drug therapy should only be used if the patient is not willing to change their lifestyle | No | 114 (86.4%) | 18 (13.6%) |
| Counselling about lifestyle interventions to prevent and control hypertension should be advised in all patients | Yes | 128 (97.0%) | 4 (3.0%) |
| All the health care professionals assisting the patient should try to achieve the same target blood pressure | Yes | 82 (62.1%) | 50 (37.9%) |
| If the blood pressure target is not achieved, more drug treatment should be sequentially added | Yes | 70 (53.0%) | 62 (47.0%) |
| **Please indicate your view about the following aspects about prescribing antihypertensive drugs relative to the other work you must do in a routine day.** | | | |
| That long acting once a day drugs are prescribed | 1-3 (out of 10 scale, 1 being the ‘highest importance’) | 105 (79.5%) | 27 (20.5%) |
| That the patient can afford the prescribed drugs | 1-3 (out of 10 scale, 1 being the ‘highest importance’) | 103 (78.0%) | 29 (22.0%) |
| That there is a reliable high-quality supply of the prescribed drugs | 1-3 (out of 10 scale, 1 being the ‘highest importance’) | 112 (84.8%) | 20 (15.2%) |
| A5D That combination tablets if available are used if more than one drug is needed | 1-3 (out of 10 scale, 1 being the ‘highest importance’) | 87 (65.9%) | 45 (34.1%) |
| That the patient agrees they will take the medications | 1-3 (out of 10 scale, 1 being the ‘highest importance’) | 74 (56.1%) | 57 (43.9%) |
| That the drug treatment plan is as simple as possible | 1-3 (out of 10 scale, 1 being the ‘highest importance’) | 91 (68.9%) | 41 (31.1%) |
| **Is it acceptable or desirable that a non-physician health care professional perform the following tasks if they had appropriate training.** | | | |
| Measure blood pressure | Desired | 50 (37.9%) | 82 (62.1%) |
| Assess cardiovascular risk | Desired | 18 (13.6%) | 114 (86.4%) |
| Counsel about lifestyle interventions to prevent and control hypertension | Desired | 45(34.1%) | 87 (65.9%) |
| Prescribe or change antihypertensive drugs according to a physician approved pathway or algorithm | Desired | 18 (13.6%) | 114 (86.4%) |

**Table 7** **Questions Regarding Attitude**

| **What are the major barriers you face in optimizing hypertension management in your clinic?** | **Physicians, n (%)** |
| --- | --- |
| Lack of your time | 22% |
| Lack of your interest | 13.6% |
| It is not important enough compared to other work that needs to be done | 26.5% |
| The patients do not think it is important | 52.2% |
| There are too many people with hypertension | 37.8% |
| Lack of clinic leadership | 38.1% |
| Lack of adequate training. | 53.8% |
| Limited skills in clinical decision making. | 54.5% |
| Limited skills in counselling. | 50.8% |
| Lack of a clinical team. | 54.6% |
| Lack of resources (e.g. blood pressure cuffs) | 19.7% |
| Lack of quality medications (irregular supply or low quality of medicines). | 45.5% |
| Patients cannot afford the lifestyle treatment | 43.2% |
| Patients cannot afford the drug treatment | 38.7% |
| Patients have difficulty accessing the clinic. | 21.2% |

**Practice (Table 8-10)**

**Table 8** **Questions Regarding Practice**

| **How often do you personally manage patients with hypertension** | **Physicians (n=132)** |
| --- | --- |
| Most days | 14 (10.6%) |
| At least weekly | 28 (21.2%) |
| At least monthly | 50 (37.9%) |
| Less than monthly | 4 0(30.3%) |
| **In one follow-up examination, how much time does your clinic usually spend for each patient with hypertension** | **Physicians (n=132)** |
| <2 min | 0 (0%) |
| 2-<4 min | 26 (19.7%) |
| 4-<6 min | 17 (12.9%) |
| 6-<8 min | 19 (14.4%) |
| 8-10 min | 20 (15.2%) |
| 10-<12 min | 17 (12.9%) |
| 12-<14 min | 2 (1.5%) |
| 14-<16 min | 3 (2.3%) |
| 16-<18 min | 4 (3.0%) |
| >20 min | 24 (18.2%) |
| **Who measures blood pressure in the clinic where you work? Mark all that apply** | **Physicians (n=132)** |
| doctor | 67.4% |
| nurse | 83.3% |
| A member of the office staff who is not a health care professional | 1.5% |

**Table 9 Questions Regarding Practice**

|  | **Correct/Desired Answer** | **Correct /Desired (%)** | **Incorrect (%)** |
| --- | --- | --- | --- |
| What type of blood pressure device do you use? | Validated Electronic Device | 100 (75.8%) | 32 (24.2%) |
| If the clinic where you work assesses cardiovascular risk how is it assessed? | A risk chart or a risk calculator | 10 (7.6%) | 122 (92.4%) |
| Does the clinic where you work have a registry of people with hypertension? | Either a paper registry that records everyone with hypertension or a computerized registry of everyone with hypertension or both | 93 (70.5%) | 40 (29.5%) |
| If you have a hypertension registry can it provide reports on? | Registry that provides all reports on patients who have not had blood pressure measured, who has missed a visit for hypertension assessment or management, who and how many have been diagnosed with hypertension, who and how many have been treated with lifestyle change and who and how many have been treated with antihypertensive drugs | 13 (9.8%) | 119 (90.2%) |
| Does the clinic where you work use a hypertension care pathway or algorithm? | Yes, we use one in all or in most patients | 25 (18.9%) | 107 (81.1%) |
| Do you follow a specific procedure or guideline for managing hypertension emergencies/urgencies? | Both yes always and yes mostly | 48 (36.4%) | 84(63.6%) |

**Table 10** **Questions Regarding Practice**

| **In what percent of adults does the clinic where you work do the following hypertension management activities?** | **Desired (91-100%)** | **Intermediate**  **(61-90%)** | **Undesired (0-60%)** |
| --- | --- | --- | --- |
| Measure blood pressure to screen for hypertension at all adult visits | 0 (0%) | 28 (21.2%) | 104 (78.8%) |
| Counsel about hypertension, its adverse effects and the need for treatment in people with hypertension | 0 (0%) | 43(32.6%) | 86 (67.4%) |
| Counsel to use a home blood pressure measurement device in people with hypertension | 0 (0%) | 52 (39.4%) | 128 (60.6%) |
| Counsel how to properly measure blood pressure at home in people with hypertension | 11 (8.3%) | 47 (35.6%) | 74 (56.1%) |
| Assess cardiovascular risk in people with hypertension | 7 (5.3%) | 37 (28%) | 88 (66.7%) |
| Counsel about lifestyle interventions to prevent and control hypertension | 0 (0%) | 56 (42.4%) | 76 (57.6%) |
| Prescribe antihypertensive drugs in people with hypertension | 57(43.2%) | 56 (42.4%) | 19 (14.4%) |
| Prescribe antihypertensive drugs based on cardiovascular risk in people with hypertension | 0 (0%) | 44(33.3%) | 88 (66.7%) |
| Counsel for antihypertensive drug therapy and adherence to drugs in people with hypertension | 0 (0%) | 31 (23.5%) | 101(76.5%) |
| Assess adherence to antihypertensive drug therapy at all visits in people with hypertension | 0 (0%) | 31 (23.5%) | 101(76.5%) |
| **In what percent of adults with hypertension does the clinic where you work recommend the following lifestyles?** | **Desired (91-100%)** | **Intermediate**  **(61-90%)** | **Undesired (0-60%)** |
| Reducing the amount of salt in the diet | 31 (23.5%) | 23 (17.4%) | 78 (59.1%) |
| Getting regular physical activity | 48 (36.4%) | 50 (37.9%) | 34 (25.8%) |
| Reducing alcohol consumption in heavy consumers | 47 (35.6%) | 10 (7.6%) | 75 (56.8%) |
| Having a healthy body weight | 48 (36.4%) | 51 (38.6%) | 33 (25.0%) |
| **In what percent of adults with usual blood pressure of 140 or more mmHg systolic or 90 mmHg or more diastolic does the clinic where you work recommend antihypertensive drug therapy** | **Desired (91-100%)** | **Intermediate**  **(61-90%)** | **Undesired (0-60%)** |
| An adult whose usual systolic blood pressure was 160 mmHg or more | 0 (0%) | 33 (25.0%) | 99 (75.0%) |
| An adult whose usual diastolic blood pressure was 100 mmHg or more | 0 (0%) | 33 (25.0%) | 99 (75.0%) |
| An adult with more than 30% 10-year risk of a cardiovascular event | 20 (15.1%) | 26 (19.7%) | 86 (65.2%) |
| An adult with 20-29% 10-year risk of a cardiovascular event | 13 (9.0%) | 22 (16.7%) | 97 (73.5%) |
| An adult with 10-19% 10-year risk of a cardiovascular event | 8 (6.1%) | 16 (12.1%) | 108 (81.8%) |
|  | **Desired (0-10%)** | **Intermediate (11-40%)** | **Undesired (41-100%)** |
| An adult with 5-10% 10-year risk of a cardiovascular event | 25 (18.9%) | 84 (63.7%) | 23 (17.4%) |
| **In what percent of adults with usual blood pressure 140 or more mmHg systolic or 90 mmHg or more diastolic who have the following conditions would the clinic where you work recommend antihypertensive drug therapy** | **Desired (91-100%)** | **Intermediate (61-90%)** | **Undesired (0-60%)** |
| Established diabetes | 17 (12.9%) | 29 (22%) | 86 (65.2%) |
| Prior heart attack | 21 (15.9%) | 57(43.2%) | 54 (40.9%) |
| Prior stoke | 40 (30.3%) | 51 (38.6%) | 41 (31.1%) |
| Ischemic heart disease | 8 (6.1%) | 73 (55.3%) | 51 (38.6%) |
| Chronic kidney disease | 21 (15.9%) | 57 (43.2%) | 54 (40.9%) |
| Aortic aneurysm | 8 (6.1%) | 57 (43.2%) | 67 (50.7%) |
| Left ventricular hypertrophy | 6 (4.5%) | 52 (39.4%) | 74 (56.1%) |
| Heart failure | 21 (15.9%) | 57(43.2%) | 54 (40.9%) |

**Table 11** **Questions Regarding Practice**

| **When do you typically schedule blood pressure follow-ups visits ?** | **Correct/Desired Answer** | **Correct /Desired (%)** | **Shorter than recommended** | **Longer than recommended** | **Not scheduled** |
| --- | --- | --- | --- | --- | --- |
| 44 yr old asymptomatic patient with a blood pressure of 136/76 mmHg | 6-12 months | 18 (13.6%) | 93 (70.5%) | - | 21 (15.9%) |
| 44 yr old undiagnosed asymptomatic patient with a blood pressure of 152/96 mmHg | 2 weeks to 1 month | 63 (47.7%) | 66(50%) | 3 (2.3%) | 0 (0%) |
| 44 yr old undiagnosed asymptomatic patient with a blood pressure of 168/108 mmHg | 2 weeks to 1 month | 31 (23.4%) | 97 (73.5%) | 3 (2.3%) | 1 (0.8%) |
| 44 yr old undiagnosed asymptomatic patient with a blood pressure of 224/112 mmHg | Immediate referral to hospital | 94 (71.2%) | - | 37 (28.0%) | 1 (0.8%) |
| 44 yr old undiagnosed patient with headaches, blurred vision and a blood pressure of 224/112 mmHg | Immediate referral to hospital | 116 (87.9%) | - | 15 (11.3%) | 1 (0.8%) |
| New patient with recent diagnosis of hypertension just starting treatment | 2 weeks to 1 month | 48 (36.4%) | 80 (60.6%) | 4 (3.0%) | 0 (0%) |
| Patient for a routine visit when blood pressure is controlled. | 3 months | 30 (22.7%) | 77 (65.9%) | 14 (10.6%) | 1 (0.8%) |
| Patient for a visit after a treatment change for uncontrolled blood pressure control | 2 weeks to 1 month | 54 (40.9%) | 64 (48.5%) | 14 (10.6%) | 0 (0%) |

**Priority (Table 12-14)**

**Table 12** **Questions Regarding Priority**

| **PR1 How do you prioritize the following hypertension management activities compared to the work you already must do in a usual day?** | **Desired (1-3 highest priority)** | **Intermediate (4-7 priority)** | **Undesired (8-10 lowest priority)** |
| --- | --- | --- | --- |
| Measure blood pressure accurately at all appropriate visits in adults to screen for hypertension | 64(48.5%) | 51(38.7%) | 17 (12.9%) |
| Accurately diagnose hypertension | 82 (62.1%) | 35 (26.5%) | 15 (11.4%) |
| Counsel about the diagnosis of hypertension, its adverse effects and need for treatment in people with hypertension | 95 (72.0%) | 21 (15.9%) | 16 (12.1%) |
| Assess cardiovascular risk in people with hypertension | 86 (65.2%) | 30 (22.7%) | 16 (12.1%) |
| Counsel about lifestyle interventions to prevent and control hypertension | 52 (39.4%) | 51 (38.6%) | 29 (22.0%) |
| Prescribe antihypertensive drugs in people with hypertension | 71 (53.8%) | 53 (40.1%) | 8 (6.1%) |
| Prescribe antihypertensive drugs in people with hypertension at high cardiovascular risk | 74 (56.1%) | 52 (39.4%) | 6(4.5%) |
| Achieve target blood pressures in people with hypertension | 83 (62.9%) | 44 (33.4%) | 5 (3.8%) |
| Counsel about antihypertensive drug therapy and adherence to drugs in people with hypertension | 81 (61.4%) | 46 (34.8%) | 5 (3.8%) |
| Assess adherence to antihypertensive drug therapy at each visit in people prescribed with antihypertensive drugs | 53 (40.2%) | 55 (41.8%) | 24 (18.2%) |
| Recommend specific interventions to overcome unique barriers people with hypertension may face in adhering to treatment | 111 (84.1%) | 21 (15.9%) | 0 (0%) |

**Table 13** **Questions Regarding Priority**

| **Please indicate the priority of performing the following aspects of accurate blood pressure measurement relative to the rest of the work you must do every day** | **Desired (1-3 highest priority)** | **Intermediate (4-7 priority)** | **Undesired (8-10 lowest priority)** |
| --- | --- | --- | --- |
| The person doing the reading has been trained and had their ability to accurately assess blood pressure tested within the last year. | 97 (73.5%) | 23 (17.4%) | 12 (9.1%) |
| A validated electronic blood pressure device is used | 100 (75.8%) | 30 (22.7%) | 2 (1.5%) |
| The patient is rested for 5 minutes in a quiet, comfortable place before measurement | 104 (78.8%) | 25 (18.9%) | 3 (2.3%) |
| An upper arm cuff that is the correct size for the patient’s arm is used | 98 (74.2%) | 32 (24.2%) | 2 (1.6%) |
| The patients arm is supported at heart level | 100 (75.7%) | 30 (22.7%) | 2 (1.6%) |
| Blood pressure readings are taken in the sitting and standing position | 71 (53.8%) | 35 (26.5%) | 26 (19.7%) |
| The blood pressure is assessed at the initial visit in both arms and the arm with the higher blood pressure used subsequently | 86 (65.1%) | 31 (23.5%) | 15 (11.4%) |
| An average of 2 or more readings are taken in the seated position and averaged in those with initial high readings | 95 (72.0%) | 28 (21.2%) | 9 (6.8%) |
| **Please indicate the priority of the following aspects of hypertension diagnosis relative to the rest of the work that must be done every day** | | | |
| The person making the diagnosis is a physician | 104 (78.8%) | 15 (11.3%) | 13 (9.9%) |
| The blood pressures were accurately measured | 119 (90.1%) | 10 (7.6%) | 3 (2.3%) |
| There were high blood pressure readings at several visits before the diagnosis of hypertension was made unless there is a hypertensive emergency or the initial blood pressure readings were very high | 98 (74.2%) | 29 (21.9%) | 5 (3.9%) |
| Blood pressure was measured ‘out of the office’ (e.g. home readings) | 69 (52.3%) | 46 (34.8%) | 17 (12.9%) |
| The patient was assessed for hypertension organ damage | 91 (68.9%) | 29 (22.0%) | 12 (9.1%) |

**Table 14** **Questions Regarding Priority**

| **Please indicate the priority of the following aspects of counselling about the diagnosis of hypertension, its adverse effects and need for lifestyle change and drug treatment relative to the work you must do in a routine day?** | **Most Desired (1-3 highest priority)** | **Intermediate Desired (4-7 priority)** | **Undesired (8-10 lowest priority)** |
| --- | --- | --- | --- |
| Ensuring family members are involved in the counselling | 40 (30.3%) | 55 (41.7%) | 37 (28.0%) |
| Ensuring written information is provided as well as verbal information | 15 (11.4%) | 65 (49.2%) | 52 (39.4%) |
| Encouraging the patient to ask questions | 91 (68.9%) | 24 (18.2%) | 17 (12.9%) |
| Questioning the patient to ensure they understand the information | 105 (79.5%) | 27 (20.5%) | 0 (0%) |
| Providing information about the prognosis and the need for treatment at a level the patient understands | 89 (67.4%) | 43 (32.6%) | 0 (0%) |
| Reassessing the patients understanding at follow up visits | 70 (53.0%) | 43(32.6%) | 19 (14.4%) |
| **Please indicate the priority of the following aspects of about assessing cardiovascular risk compared to the work you already must do in a usual day.** | **Most Desired (1-3 highest priority)** | **Intermediate Desired (4-7 priority)** | **Undesired (8-10 lowest priority)** |
| Cardiovascular risk is assessed in all people with hypertension | 7 (5.3%) | 37 (28.0%) | 88 (66.7%) |
| Cardiovascular risk is assessed using an objective tool such as a risk chart or computer program | 10 (7.6%) | 85 (64.4%) | 37 (28.0%) |
| The cardiovascular risk tool is calibrated to the Chinese population | 10 (7.6%) | 85 (64.4%) | 37 (28.0%) |
| People with established diabetes are assessed as being at high risk | 44 (33.3%) | 78 (59.1%) | 10 (7.6%) |
| People with a prior stroke, heart attack or other cardiovascular disease are assessed as being at high risk | 87 (65.9%) | 41 (31.14%) | 4 (3.0%) |
| **Please indicate the priority of the following aspects about counselling for antihypertensive drug therapy and adherence to drugs relative to the work you must do in a routine day** | **Most Desired (1-3 highest priority)** | **Intermediate Desired (4-7 priority)** | **Undesired (8-10 lowest priority)** |
| That the benefits of the treatment and the low probability of adverse effects is understood by the patient | 108 (81.8%) | 20 (15.2%) | 4 (3.0%) |
| That serious and more common adverse effects are explained to the patient, the patient knows to report problems and that the treatment plan can be revised if needed is understood by the patient | 101 (76.5%) | 23 (17.4%) | 8 (6.1%) |
| That the importance of adherence to lifelong therapy even when hypertension is controlled is understood by the patient | 96 (72.7%) | 26 (16.7%) | 10 (7.6%) |
| That the patient is telephoned after treatment is started to assess adherence and answer questions they have | 89 (67.4%) | 106 (25.8%) | 9 (6.8%) |
| That close follow-up is scheduled until blood pressure is controlled and then regular follow-up visits are scheduled | 100 (75.8%) | 25 (18.9%) | 7 (5.3%) |
| That potential barriers to lifestyle and drug adherence are asked about and solutions developed with the patient | 53 (40.1%) | 55 (41.6%) | 24 (18.2%) |
| The patient is able to afford and implement the therapeutic plan | 103 (78.0%) | 25 (19.0%) | 4 (3.0%) |
| That training is provided to the patient to incorporate lifestyle and drug treatments into their daily routine | 71 (53.8%) | 55 (41.7%) | 6 (4.5%) |
| That the whole healthcare team (physician, nurse, pharmacists etc.) emphasize the importance of controlling hypertension and adherence to lifestyle and drug treatment | 96 (72.7%) | 22 (16.7%) | 14 (10.6%) |
| That dosettes and other adherence devices are recommended in people prescribed complex drug treatments | 83 (62.9%) | 38 (28.8%) | 11 (8.3%) |
| The patient and health care professional agree with the therapeutic plan | 97 (73.5%) | 27 (20.4%) | 8 (6.1%) |
| How do you prioritize having a registry with regular reports on hypertension diagnosis, treatment and control? | 62 (47.0%) | 31 (23.5%) | 39 (29.5%) |
| How do you prioritize using a treatment pathway or algorithm to manage hypertension? | 25 (18.9%) | 48 (36.4%) | 59 (54.7%) |

**Confidence (Table 15-16)**

**Table 15** **Question Regarding Confidence On Prescribing Antihypertensive Medication**

|  | **3 or more drugs (%)** | **2 drugs (%)** | **1 drug (%)** |
| --- | --- | --- | --- |
| What is the maximum number of antihypertensive drugs you feel confident in prescribing in a single patient with hypertension? | 44 (33.3%) | 76 (57.6%) | 12 (9.1%) |

**Table 16** **Questions Regarding Confidence**

|  | **Desired(8-10) (%)** | **Intermediate(4-7) (%)** | **Undesired (1-3) (%)** |
| --- | --- | --- | --- |
| Without additional training how confident are you that you can implement and use a registry that has regular reports on hypertension diagnosis, treatment and control? | 44 (33.3%) | 52 (39.4%) | 36 (27.3%) |
| Without additional training how confident are you that you can implement and use a treatment algorithm or pathway in your clinic? | 25 (18.9%) | 62 (47.0%) | 45 (34.1%) |
| **Without additional training how confident do you feel in your ability to optimally perform the following activities in diagnosing and managing hypertension?** | **Desired (8-10) (%)** | **Intermediate (4-7) (%)** | **Undesired (1-3) (%)** |
| Measure blood pressure accurately at all appropriate visits in adults to screen for hypertension | 53 (40.2%) | 45 (34.1%) | 34 (25.8%) |
| Accurately diagnose hypertension | 47 (35.6%) | 43 (32.6%) | 42 (31.8%) |
| Counsel about hypertension and its adverse effects and the need for treatment | 52 (39.4%) | 44 (33.3%) | 36 (27.3%) |
| Counsel about lifestyle interventions to prevent and control hypertension | 51 (38.6%) | 38 (28.8%) | 43 (32.6%) |
| Prescribe antihypertensive drugs | 48 (36.4%) | 40 (30.3%) | 44 (33.3%) |
| Achieve target blood pressures with lifestyle and or drug treatment | 47 (35.6%) | 51 (38.6%) | 34 (25.8%) |
| Counsel about antihypertensive drug therapy and adherence to drugs | 51 (38.6%) | 45 (34.1%) | 36 (27.3%) |
| Assess adherence to antihypertensive drug therapy at each visit in people prescribed antihypertensive drugs | 31 (23.5%) | 51 (38.6%) | 50(37.9%) |
